# Modern Highland Problems

**Published:** 1906-11-10

**Authors:** Lachlan Grant

**Affiliations:** Ardarroch, Ballachulish, Glencoe


### Editor's Letter-Box.

[Our Correspondents are reminded that prolixity is a great bar to publication, and that brevity of style and conciseness of statement greatly facilitate early insertion.]

### MODERN HIGHLAND PROBLEMS.

*To the Editor of* The Hospital.

Sir,—I must thank you for notice of my paper on "Modern Highland Problems" in this week's Hospital. Your remarks were very much to the point, and cannot fail to make for progress. The centripetal movement of the people towards the cities, which is occurring in every country, is telling heavily on our Highland population. I think medical papers should take up this question from the public health point of view.

Here we have many hardy Highlanders willing to remain on and till the soil; and, instead of being encouraged to do so, they are in many places being cleared off, the land being put under deer, and the glens left silent.

We all hope the present Land Bill will pass. In my opinion it will help to remedy many of the evils the Highlands now suffer from.

I am, yours sincerely,

Ardarroch, Ballachulish, Glencoe, November 2, 1906. Lachlan Grant.

